# Diagnostic value of CEACAM6 and HE4 in pleural fluid for malignant pleural effusion

**DOI:** 10.1080/07853890.2025.2489748

**Published:** 2025-04-15

**Authors:** Jie Li, Liyuan Lin, Shengrui Yang, Yuan Mu, Lixia Zhang, Haoyu Ruan, Jian Xu

**Affiliations:** a Department of Laboratory Medicine, The First Affiliated Hospital of Nanjing Medical University, Nanjing, China; b Branch of National Clinical Research Center for Laboratory Medicine, Nanjing, China

**Keywords:** CEACAM6, HE4, malignant pleural effusion, diagnosis, sensitivity, specificity, AUC

## Abstract

**Objective:**

This study aimed to assess the diagnostic performance of carcinoembryonic antigen-related adhesion molecule 6 (CEACAM6) and human epididymis protein 4 (HE4) in pleural fluid for the detection of malignant pleural effusion (MPE).

**Materials and methods:**

In this prospective study, pleural levels of CEACAM6 and HE4 were measured in two independent cohorts. The test cohort included 182 patients with exudative pleural effusions (123 malignant and 59 benign), and the validation cohort comprised 117 patients with exudative pleural effusions (65 malignant and 52 benign). Receiver operating characteristic (ROC) curves were used to assess the diagnostic performance of CEACAM6 and HE4 for MPE.

**Results:**

Both CEACAM6 and HE4 levels were significantly elevated in MPE compared to benign pleural effusion (BPE) in both cohorts (*p* < .001). In the test cohort, CEACAM6 and HE4 demonstrated areas under the curve (AUC) values of 0.862 and 0.826, respectively. The combination of CEACAM6 and HE4 yielded a higher AUC of 0.938 compared to either marker alone. In the validation cohort, both CEACAM6 (AUC = 0.811) and HE4 (AUC = 0.721), along with their combination (AUC = 0.834), exhibited strong diagnostic performance for MPE. Notably, in cytology-negative cases, the combination of CEACAM6 and HE4 also demonstrated a favourable diagnostic efficacy, with an AUC of 0.800. The addition of CEA to the CEACAM6/HE4 combination further improved the AUC to 0.819 (*p* = .04).

**Conclusions:**

Pleural CEACAM6 and HE4 are promising biomarkers for distinguishing MPE from BPE. Their combination improves diagnostic accuracy, offering a valuable tool for MPE diagnosis, especially in challenging cases with cytology-negative pleural effusion.

## 1. Introduction

Malignant pleural effusion (MPE) is a common complication of malignancies, and it is closely associated with a poor prognosis [1]. Generally, patients with MPE have a mean survival of only 3–12 months. However, the diagnosis of MPE remains challenging. Cytologic examination of pleural effusion (PE) obtained via thoracentesis is a standard method for the diagnosis of MPE but has a poor sensitivity of 30–60% [2,3]. Therefore, there is a pressing need for new diagnostic methods to improve accuracy in identifying MPE. Tumour markers have been explored extensively to distinguish between malignant and benign PEs, such as carcinoembryonic antigen (CEA), carbohydrate antigen 125 (CA125), carbohydrate antigen 19-9 (CA19-9), neuron-specific enolase (NSE) and cytokeratin 19 fragments (CYFRA21-1) [4]. In recent years, many new biomarkers such as vascular endothelial growth factor (VEGF) [5] and periostin [6], have also shown promise for MPE diagnosis. However, the diagnostic performance of these markers needs to be further improved.

CEACAM6, also known as the cluster of differentiation 66c (CD66c), belongs to the CEACAM family [7]. CEACAM6 is frequently overexpressed in some types of cancer, such as pancreatic [8], lung [9] and colorectal cancers [10], and is considered as a promising tumour marker [11]. It is involved in the development of tumours, including the promotion of tumour proliferation, metastases and angiogenesis, the inhibition of tumour apoptosis, and the induction of drug resistance [12–14]. A previous study has expounded upon the diagnostic efficacy of CEACAM6 regarding lung adenocarcinoma (LUAD) associated MPE [15]. In our study, we tried to investigate the diagnostic value of CEACAM6 in MPE and lung cancer-related MPE (LC-MPE).

Human epididymis protein 4 (HE4) is an important biomarker for ovarian cancer [16] and is also a useful biomarker for lung cancer (LC) [17]. The elevated serum HE4 levels can sometimes indicate LC metastasis, especially LC bone metastasis [18]. And it is considered to have potential value in the differential diagnosis of MPE [19,20] and LC-MPE [21]. In our previous study, elevated levels of CEACAM6 or HE4 were found in the cerebrospinal fluid (CSF) of LUAD patients with leptomeningeal metastases (LMs). The good performance in distinguishing LM from non-LM was achieved by the CSF levels of HE4 and CEACAM6 [22,23]. Furthermore, the combination of CEACAM6 and HE4 could serve as a better CSF biomarker for LUAD-LM. However, the potential of the combination of HE4 and CEACAM6 in PE as biomarkers for MPE diagnosis remains unclear. In this study, we intend to analyse the CEACAM6 and HE4 levels in the PEs to determine their diagnostic value for MPE.

## 2. Materials and methods

### 2.1. Patients

**Figure 1.**
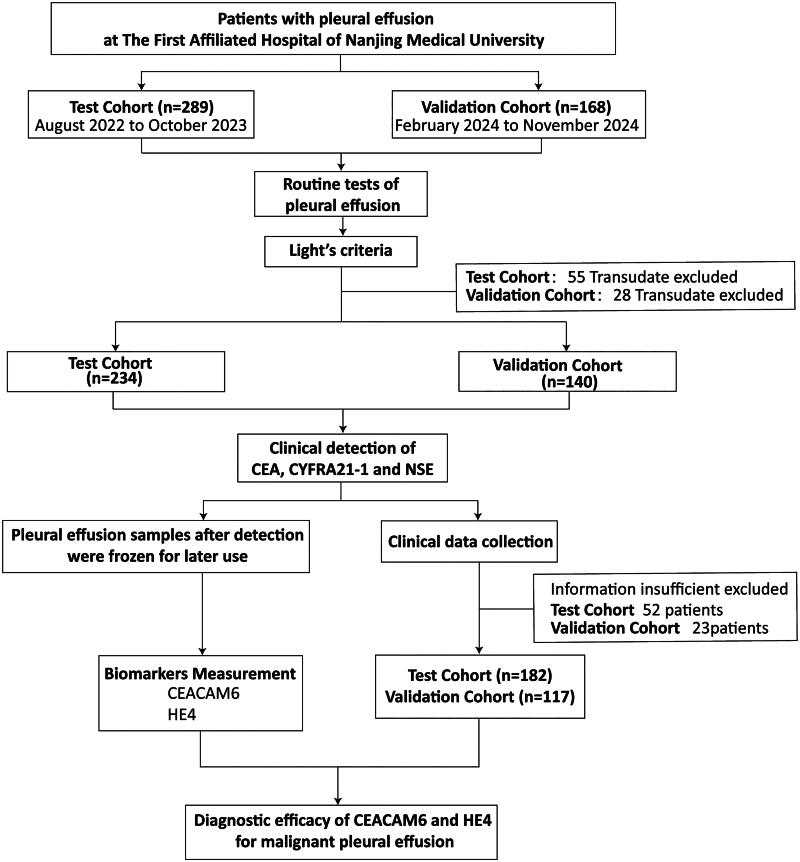
Study workflow.

This study was a prospective diagnostic accuracy study, and the PE samples were all collected from the First Affiliated Hospital of Nanjing Medical University. Between August 2022 and October 2023, 289 individuals with PE were sequentially recruited as a test cohort. Subsequently, from February 2024 to November 2024, an additional cohort comprising 168 PE patients was consecutively enrolled to serve as a validation cohort. According to the Chinese expert consensus on the diagnosis of PE [24] and Light’s criteria, 55 patients with transudate and an additional 52 patients with incomplete information were excluded from the test cohort, while 28 transudate cases and 23 cases with incomplete data were excluded from the validation cohort. As a result, the test cohort consisted of 182 patients (123 MPE and 59 benign pleural effusion (BPE)), and the validation cohort included 117 patients (65 MPE and 52 BPE). MPE was diagnosed by identifying malignant cells in multiple cytology tests or biopsies. For PEs with initially negative cytology results, the effusion is considered malignant if tumour cells are detected in subsequent samples or if histological examination of a pleural biopsy confirms malignancy. The BPE group included parapneumonic PE (diagnosed with acute febrile illness, purulent sputum and pulmonary infiltrates, in the absence of malignancy or transudate-causing diseases) and tuberculous pleurisy (diagnosed by positive *Mycobacterium tuberculosis* culture or typical epithelioid cell granuloma in pleural biopsy). Clinical data, including age, gender, and clinical and histological grades were recorded. The study workflow is illustrated in Figure 1. This research was approved by the Research and Ethics Committee of the First Affiliated Hospital of Nanjing Medical University (2022-SRFA-037), in accordance with the Declaration of Helsinki and its subsequent amendments. Participants provided written informed consent to take part in the study.

### 2.2. Sample collection and analysis

PE samples were collected from patients with PEs at the First Affiliated Hospital of Nanjing Medical University. Following routine tests and tumour marker detection (CEA, NSE and CYFRA21-1), the remaining samples were centrifuged at 3000 × *g* for 10 min at 4 °C. The supernatants were collected and stored at −80 °C for subsequent determination of CEACAM6 and HE4 levels. CEACAM6 was detected by ELISA (Sino Biological, Beijing, China) according to the manufacturer’s instructions. HE4 was measured using chemiluminescent immunoassay (CLIA) on the Roche Cobas e602 system (Basel, Switzerland). The specimens for PE cytology examination and the PE samples we collected were taken at the same time point. The relevant clinical data were obtained from medical records.

### 2.3. Statistical analysis

Statistical analyses were carried out using SPSS (version 26.0, Chicago, IL), MedCalc (version 20.0.4, New York, NY) and GraphPad Prism 8 (version 8.0.2, La Jolla, CA) for plotting. Categorical variables were expressed as counts and percentages and compared using the Chi-square test or Fisher’s exact test. Continuous variables, which exhibited a non-normal distribution as determined by the Kolmogorov–Smirnov test, were expressed as median values with interquartile ranges [median (*Q*25-*Q*75)]. The Mann–Whitney *U*-test was used to compare continuous variables between two groups. Receiver operating characteristic (ROC) curve analysis was employed to calculate the curve (AUC), sensitivity, specificity, negative predictive value (NPV), positive predictive value (PPV), negative likelihood ratio (−LR), positive likelihood ratio (+LR) and the cut-off value to assess the diagnostic performance of CEACAM6 or HE4, both individually and in combination. The Youden index was derived by combining sensitivity and specificity, and the optimal threshold corresponding to the maximum Youden index was identified. Logistic regression was used to assess the combination of multiple tumour markers. The cut-off values derived from this cohort were then applied to both the validation and cytology-negative cohorts. We classified the samples in the validation cohort into positive and negative groups for ROC analysis against the actual diagnostic results and performed the same analysis for the cytology-negative cohort. DeLong’s test was used to assess the statistical significance between the two ROC curves.

## 3. Results

### 3.1. Clinical characteristics of patients

The clinical characteristics of patients in the test and validation cohorts are summarized in Table 1. No significant differences were observed between the MPE and BPE groups in terms of age or gender in both cohorts. In the test cohort, no significant differences were found in laboratory parameters, including pLDH, sLDH, pTP, sTP and their respective ratios, between the MPE and BPE groups. In the validation cohort, while no significant differences in pLDH or sTP were noted between the two groups, statistically significant differences were observed in sLDH and pTP levels (*p* = .036 and *p* = .035, respectively).

**Table 1.** Clinical characteristics of patients with PE.

	MPE	BPE	*p* Value
*Test cohort* (*n* = 182)			
Age, years	64 (56–71)	68 (56–78)	.265
Sex, %			
Male	74 (60.16%)	41 (69.49%)	.222
Female	49 (39.84%)	18 (30.51%)	
Laboratory parameters			
pLDH (U/mL)	309.0 (203.0–562.0)	271.0 (179.8–523.3)	.143
sLDH (U/mL)	216.0 (176.0–265.0)	222.0 (1177.0–260.5)	.630
pLDH/sLDH	1.3 (0.8–2.4)	1.1 (0.7–2.6)	.194
pTP (g/L)	40.2 (35.0–47.6)	44.5 (37.8–48.1)	.467
sTP (g/L)	62.6 (58.7–68.4)	61.3 (56.4–64.9)	.183
pTP/sTP	0.6 (0.5–0.8)	0.7 (0.6–0.8)	.504
Diagnosis	LC-MPE (*n* = 102)	PPE (*n* = 48)	
	Other MPE (*n* = 21)	TPE (*n* = 11)	
*Validation cohort* (*n* = 117)			
Age, years	67 (60–74)	65.5 (57–78)	.988
Sex, %			
Male	30 (46.15%)	30 (57.69%)	.215
Female	35 (53.85%)	22 (42.31%)	
Laboratory parameters			
pLDH (U/mL)	305 (180.5–861.5)	225 (105.3–434.8)	.552
sLDH (U/mL)	246.0 (203.0–331.5)	222.5 (178.3–277)	.036
pLDH/sLDH	1.18 (0.74–2.71)	0.93 (0.53–2.07)	.004
pTP (g/L)	42.3 (35.4–47.4)	34.4 (20.3–45.4)	.035
sTP (g/L)	63.4 (56.9–69.1)	58.3 (53.5–63.5)	.885
pTP/sTP	0.67 (0.57–0.73)	0.59 (0.39–0.72)	.015
Diagnosis	LC-MPE (*n* = 38)	PPE (*n* = 45)	
	Other MPE (*n* = 27)	TPE (*n* = 7)	

pLDH: LDH expression level in PE; sLDH: LDH expression level in serum; pTP: TP expression level in PE; sTP: TP expression level in serum; LC-MPE: lung cancer-related MPE; other MPE: other malignancies-related MPE; PPE: parapneumonic PE; TPE: tuberculous PE.

Measures expressed as medians (interquartile range).

### 3.2. Pleural levels of CEACAM6 and HE4 were elevated in MPE in both the test and validation cohorts

**Figure 2.**
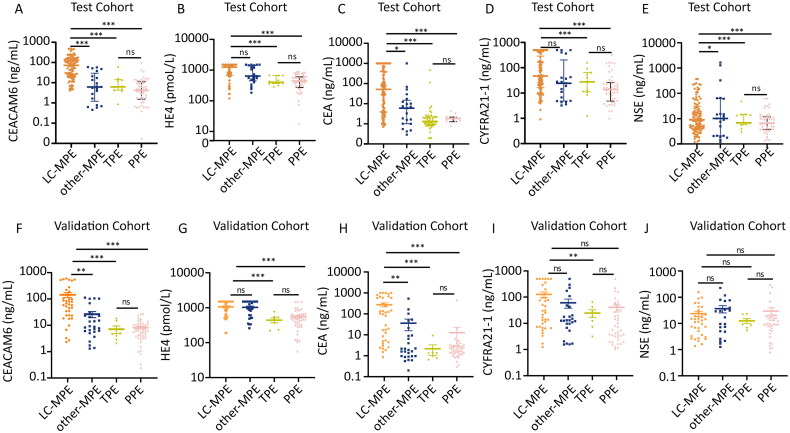
Levels of biomarkers in PEs. (A–E) Pleural levels of CEACAM6, HE4, CEA, CYFRA21-1 and NSE in each subgroup of MPE and BPE groups in test cohort. (F–J) Pleural levels of CEACAM6, HE4, CEA, CYFRA21-1 and NSE in each subgroup of MPE and BPE groups in validation cohort. The *Y*-axis is presented on a logarithmic scale. *p < .05, **p < .01, ***p < .001, Mann–Whitney *U*-test.

The pleural levels of CEACAM6, HE4 and clinically used tumour markers, including CEA, CYFRA21-1 and NSE, are presented in Table 2 and Figure 2. In the test cohort, the levels of CEACAM6 and HE4 were significantly higher in the MPE group compared to the BPE group (both *p* < .001). Additionally, the levels of CEA, CYFRA21-1 and NSE were also elevated in the MPE group (all *p* < .01). When the MPE group was subdivided into LC-MPE and other MPE, CEACAM6 levels in LC-MPE were notably higher than in other MPE.

**Table 2.** The levels of CEACAM6 and HE4 in PEs (median/IQR).

	MPE	BPE	*p* Value
*Test cohort* (*n* = 182)			
CEACAM6 (ng/mL)	49.31 (15.56–129.28)	4.69 (1.69–12.10)	<.001
HE4 (pmol/L)	1119.00 (624.30–1500.00)	434.80 (292.20–600.50)	<.001
CEA (ng/mL)	28.90 (3.27–270.70)	1.35 (1.01–2.23)	<.001
CYFRA21-1 (ng/mL)	40.16 (12.39–129.28)	14.19 (5.57–32.07)	<.001
NSE (ng/mL)	9.06 (3.88–30.93)	6.84 (3.81–13.395)	<.01
*Validation cohort* (*n* = 117)			
CEACAM6 (ng/mL)	29.04 (8.02–100.38)	5.88 (3.49–5.88)	<.001
HE4 (pmol/L)	1046.00 (607.75–1500)	487.20 (329.40–655.68)	<.001
CEA (ng/mL)	9.29 (1.90–141.20)	1.78 (1.26–3.06)	<.001
CYFRA21-1 (ng/mL)	19.05 (6.76–115)	7.85 (3.12–43.30)	<.05
NSE (ng/mL)	13.00 (3.64–32.40)	9.50 (6.39–19.95)	>.05

In the validation cohort, patients with MPE also showed higher concentrations of CEACAM6 and HE4 compared to those with BPE (both *p* < .001) (Table 2). Furthermore, CEA and CYFRA21-1 levels were elevated in the MPE group compared to the BPE group (both *p* < .05). Similar to the test cohort, CEACAM6 levels in LC-MPE were significantly higher than those in other MPE (*p* < .01).

### 3.3. The diagnostic performance of pleural CEACAM6 and HE4 for MPE in test cohort

**Figure 3.**
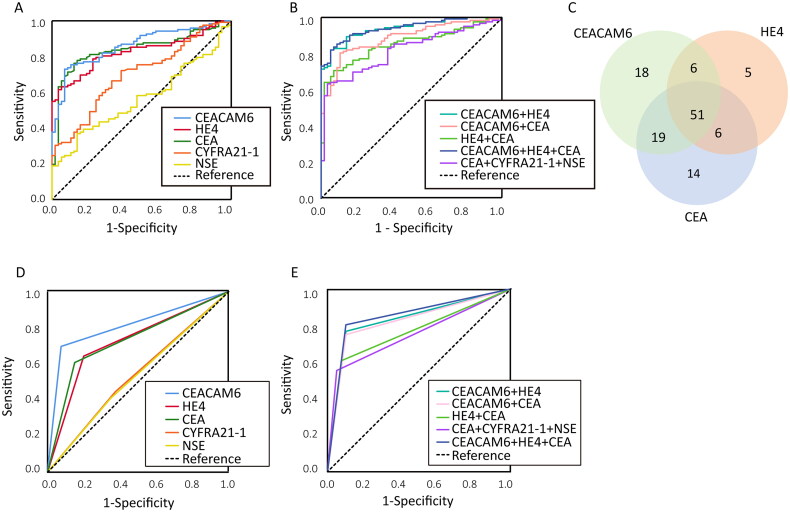
The diagnostic efficacy of individual biomarkers and their combination for differentiating MPE from BPE in test cohort. (A) The ROC curve of test cohort for each of the biomarkers CEACAM6, HE4, CEA, CYFRA21-1 and NSE individually. (B) The ROC curve of test cohort for the combination of these biomarkers. (C) Venn diagram showing cases with positive results for CEACAM6, HE4 and CEA in the MPE group in test cohort. (D) The ROC curve of validation cohort for each of the biomarkers CEACAM6, HE4, CEA, CYFRA21-1 and NSE individually. (E) The ROC curve of validation cohort for the combination of these biomarkers.

To evaluate the diagnostic performance of CEACAM6 and HE4 for MPE in the test cohort, ROC curves were generated (Figure 3(A,B)). The AUC for CEACAM6 was 0.862 (0.804–0.909), demonstrating its ability to distinguish MPE from BPE. At a cut-off value of 17.65 ng/mL, CEACAM6 showed a sensitivity of 73.2% (64.4–80.8) and a specificity of 93.2% (83.5–98.1). For HE4, the AUC was 0.826 (0.763–0.878), with a cut-off value of 809.80 pmol/L, yielding a sensitivity of 61.8% (52.6–70.4) and a specificity of 96.6% (88.3–99.6) (Table 3). The AUC values of classical tumour markers CEA, CYFRA21-1 and NSE were 0.841 (0.779–0.891), 0.699 (0.627–0.764) and 0.566 (0.490–0.639), respectively. Both CEACAM6 and CEA exhibited superior sensitivity, while CEACAM6 and HE4 showed the highest specificity among all markers, highlighting their diagnostic utility for MPE (Table 3).

**Table 3.** Diagnostic efficacy of individual pleural biomarkers and their combinations for identifying MPE in test cohort.

Variables	Cut-off value	Sensitivity (95%CI)	Specificity (95%CI)	+LR (95%CI)	−LR (95%CI)	AUC (95%CI)	PPV (95%CI)	NPV (95%CI)
*Single biomarker*								
CEACAM6	17.65 (ng/mL)	73.2% (64.4–80.8)	93.2% (83.5–98.1)	10.8 (4.2–28.0)	0.3 (0.2–0.4)	0.862 (0.804–0.909)	95.7% (92.1–97.8)	62.5% (55.4–69.1)
HE4	809.80 (pmol/L)	61.8% (52.6–70.4)	96.6% (88.3–99.6)	18.2 (4.6–71.7)	0.4 (0.3–0.5)	0.826 (0.763–0.878)	97.4% (93.6–98.9)	54.8% (49.2–60.4)
CEA	3.21 (ng/mL)	75.6% (67.0–82.9)	88.1% (77.1–95.1)	6.4 (3.2–12.9)	0.3 (0.2–0.4)	0.841 (0.779–0.891)	93.0% (89.0–95.6)	63.4% (55.8–70.4)
CYFRA21-1	23.58 (ng/mL)	65.9% (56.8–74.2)	67.8% (54.4–79.4)	2.0 (1.4–3.0)	0.5 (0.4–0.7)	0.699 (0.627–0.764)	81.0% (76.2–85.0)	48.8% (42.0–55.7)
NSE	15.1 (ng/mL)	36.9% (28.3–46.1)	86.0% (74.2–93.7)	2.6 (1.3–5.2)	0.7 (0.6–0.9)	0.566 (0.490–0.639)	84.6% (77.0–90.0)	39.5% (35.9–43.2)
*Biomarker combination*								
CEACAM6 + HE4	–	82.1% (74.2–88.4)	94.9% (85.9–98.9)	16.2 (5.3–48.8)	0.2 (0.1–0.3)	0.938 (0.893–0.968)	97.1% (94.0–98.6)	71.8% (63.5–78.8)
CEACAM6 + CEA	–	80.5% (72.4–87.1)	89.8% (79.2–96.2)	7.9 (3.7–17.0)	0.2 (0.2–0.3)	0.887 (0.832–0.929)	94.3% (90.6–96.6)	68.8% (60.6–76.1)
HE4 + CEA	–	70.7% (61.9–78.6)	93.2% (83.5–98.1)	10.4 (4.0–27.1)	0.3 (0.2–0.4)	0.850 (0.790–0.898)	95.6% (91.8–97.7)	60.4% (53.6–66.9)
CEACAM6 + HE4 + CEA	–	82.1% (74.2–88.4)	94.9% (85.9–98.9)	16.2 (5.3–48.8)	0.2 (0.1–0.3)	0.941 (0.896–0.970)	97.1% (94.0–98.7)	71.8% (63.5–78.8)
CEA + CYFRA21-1 + NSE	–	63.1% (53.9–71.7)	96.5% (87.9–99.6)	17.9 (4.6–70.7)	0.4 (0.3–0.5)	0.818 (0.753–0.871)	97.4% (96.6–98.9)	55.7% (49.8–61.3)

A Venn diagram of positive test results for CEACAM6, HE4 and CEA in the MPE group (test cohort) is shown in Figure 3(C). A total of 51 patients tested positive for all three biomarkers, 70 patients for both CEA and CEACAM6, 57 for both CEACAM6 and HE4, and 57 for both CEA and HE4. These findings emphasized the complementary roles of these biomarkers and the values of utilizing multiple indicators in MPE diagnosis.

The combined diagnostic performance of CEACAM6 and HE4 for MPE was evaluated (Table 3, Figure 3(B)). This combination outperformed single markers, showing higher sensitivity [82.1% (74.2–88.4)] and specificity [94.9% (85.9–98.9)], with an AUC of 0.938 (0.893–0.968). The addition of CEA to the combination of CEACAM6 and HE4 did not significantly improve the AUC value (0.941 (0.896–0.970), *p* = .50) (Table 3, Supplementary Table S1, Fig. S2A).

### 3.4. Validation of the diagnostic value of pleural CEACAM6 and HE4 for MPE in an independent cohort

A validation cohort was used to confirm the diagnostic value of PE CEACAM6 and HE4 for MPE (Table 4, Figure 3(D,E)). As expected, both CEACAM6 and HE4 demonstrated good diagnostic efficacy. For CEACAM6, using a cut-off of 17.65 ng/mL determined in the test cohort, the AUC for differentiating MPE from BPE in the validation cohort was 0.811 (0.722–0.899). For HE4, using a cut-off of 809.80 pmol/L, the AUC was 0.721 (0.617–0.825). The AUCs of CEA, CYFRA21-1 and NSE were 0.729 (0.626–0.831), 0.536 (0.419–0.653) and 0.530 (0.413–0.648), respectively (Table 4, Figure 3(D)).

**Table 4.** Validation of the diagnostic significance of PE CEACEAM6 and HE4 in MPE within an independent cohort.

Variables	Sensitivity (95%CI)	Specificity (95%CI)	+LR (95%CI)	−LR (95%CI)	AUC (95%CI)	PPV (95%CI)	NPV (95%CI))
*Single biomarker*							
CEACAM6	64.6% (51.8–76.1)	92.3% (81.5–97.9)	8.9 (3.2–21.9)	0.4 (0.3–0.5)	0.811 (0.722–0.899)	91.3% (80.1–96.5)	67.6% (59.8–74.5)
HE4	67.8% (54.9–78.8)	82.7% (69.7–91.8)	3.9 (2.1–7.3)	0.4 (0.3–0.6)	0.721 (0.617–0.825)	83% (72.5–90.1)	67.2% (58.5–74.8)
CEA	63.1% (50.2–74.7)	76.9% (63.2–87.5)	2.7 (1.6–4.6)	0.5 (0.3–0.7)	0.729 (0.626–0.831)	77.4% (66.8–85.3)	62.5% (54.0–70.3)
CYFRA21-1	46.6% (33.1–60.1)	63.6% (47.8–77.6)	1.3 (0.8–2.1)	0.8 (0.6–1.2)	0.536 (0.419–0.653)	62.8% (51.1–73.1)	47.5% (39.4–55.6)
NSE	42.4% (29.6–55.9)	63.1% (47.5–76.8)	1.2 (0.7–1.9)	0.9 (0.7–1.2)	0.530 (0.413–0.648)	59.5% (47.6–70.4)	46% (38.5–53.8)
*Biomarker combination*							
CEACAM6 + HE4	76.9% (64.8–86.5)	92.3% (81.5–97.9)	10.0 (3.9–25.9)	0.3 (0.2–0.4)	0.834 (0.749–0.919)	92.6% (82.8–97.0)	76.2% (67.1–83.4)
CEACAM6 + CEA	69.0% (56.6–80.1)	88.5% (76.6–95.6)	6.0 (2.8–13.0)	0.4 (0.2–0.5)	0.825 (0.738–0.912)	88.2% (77.6–94.2)	69.7% (61.2–77.0)
HE4 + CEA	63.1% (50.2–74.7)	92.3% (81.5–97.9)	8.2 (3.1–21.4)	0.4 (3.1–21.4)	0.766 (0.670–0.862)	91.1% (79.7–96.4)	66.7% (59.0–73.5)
CEACAM6 + HE4 + CEA	80.0% (68.2–88.9)	90.4% (79.0–96.8)	8.3 (3.6–19.3)	0.2 (0.1–0.4)	0.852 (0.770–0.934)	91.2% (81.8–96.0)	78.3% (68.8–85.6)
CEA + CYFRA21-1 + NSE	55.4% (41.5–68.7)	95% (83.1–99.4)	11.1(2.8–43.6)	0.5 (0.3–0.6)	0.752 (0.655–0.749)	93.9% (79.7–98.4)	60.3% (53.0–67.2)

The combination of CEACAM6 and HE4 exhibited superior diagnostic performance, with an AUC of 0.834 (0.749–0.919), outperforming individual biomarkers and all other pairwise combinations (Table 4, Figure 3(E)). Incorporating CEA further increased the AUC to 0.852 (*p* = .75) (Table 4, Supplementary Table S1, Fig. S2B). These findings further validate the diagnostic utility of CEACAM6 and HE4 for MPE.

### 3.5. The diagnostic performance of pleural CEACAM6 and HE4 for LC-MPE in both the test and validation cohorts

Since LC-MPE is the most common form of MPE, we further assessed the diagnostic value of pleural CEACAM6 and HE4 in differentiating LC-MPE from BPE using ROC analysis (Supplementary Table S2, Fig. S1). In the test cohort, the AUC of CEACAM6 was 0.922 (0.869–0.958), with a sensitivity of 82.7% (73.6–89.2) and specificity of 93.2% (83.5–98.1). For HE4, the AUC was 0.847 (0.782–0.899), with sensitivity of 67.7% (57.7–76.6) and specificity of 94.9% (85.9–98.9) (Supplementary Table S2, Fig. S1A). The combination of CEACAM6 and HE4 further improved diagnostic performance, achieved an AUC to 0.967 (0.926–0.989), surpassing both the individual biomarkers and all other two-marker combinations (Supplementary Table S2, Fig. S1B). The addition of CEA to the CEACAM6/HE4 combination resulted in a slight increase in AUC to 0.970 (0.930–0.990), though this change was not statistically significant (*p* = .39) (Supplementary Tables S1 and S2, Fig. S2C).

In the validation cohort, using the same cut-off values for CEACAM6 (17.65 ng/mL) and HE4 (780.10 pmol/L) as in the test cohort, ROC analysis showed an AUC of 0.866 (0.778–0.929) for CEACAM6, 0.756 (0.654–0.840) for HE4, and 0.876 (0.789–0.936) for the combination of CEACAM6 and HE4 (Supplementary Table S3, Fig. S1C,D). The validation cohort further confirmed the diagnostic value of HE4 and CEACAM6 combination for LC-MPE. With the addition of CEA, the AUC was improved to 0.925 (0.849–0.970) (*p* = .08) (Supplementary Tables S1 and S3, Fig. S2D).

### 3.6. The diagnostic performance of CEACAM6 and HE4 for MPE in cytology-negative cases

**Figure 4.**
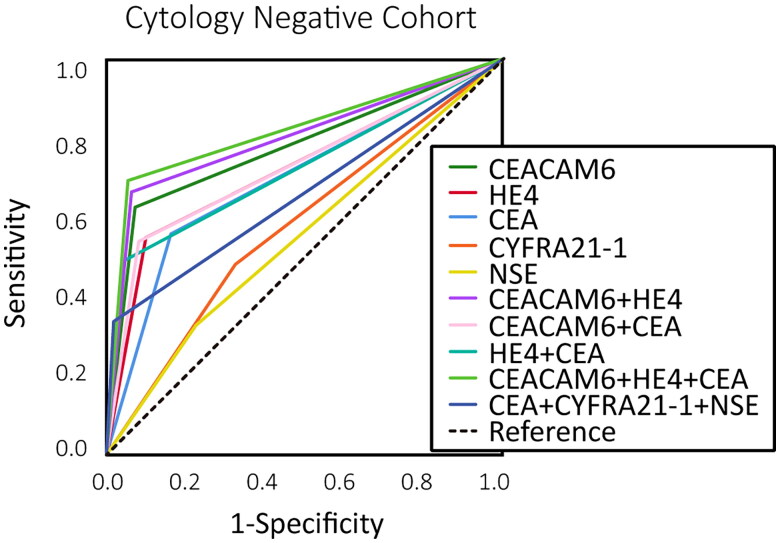
The ROC curve for differentiating MPE from BPE of cytology negative cohort.

We further evaluated the diagnostic value of CEACAM6 and HE4 in patients with negative cytology results. This cohort consisted of 211 patients, 111 of whom were diagnosed with BPE and 100 with MPE. Among the 100 MPE, 60 were from the test cohort, and 40 from the validation cohort (Supplementary Table S4). The cut-off values derived from the test cohort were applied to the cytology-negative cohort for ROC analysis. Our results demonstrated that the combination of CEACAM6 and HE4 exhibited strong diagnostic performance, with a sensitivity of 66.4% (56.4–75.3), specificity of 93.7% (87.4–97.4) and an AUC of 0.800 (0.740–0.851) (Table 5, Figure 4). Furthermore, the inclusion of CEA in the combination of CEACAM6 and HE4 increased the AUC to 0.819 (0.761–0.868) (*p* = .04) (Table 5, Supplementary Table S1, Fig. S2E). These findings further supported the diagnostic value of combining CEACAM6, HE4 and CEA for MPE in cytology-negative cases.

**Table 5.** Diagnostic efficacy of individual pleural biomarkers and their combinations for identifying MPE in cytology negative cohort.

Variables	Sensitivity (95%CI)	Specificity (95%CI)	+LR (95%CI)	−LR (95%CI)	AUC (95%CI)	PPV (95%CI)	NPV (95%CI)
*Single biomarker*							
CEACAM6	62.5% (52.5–71.8)	92.8% (86.3–96.8)	8.7 (4.4–17.2)	0.4 (0.3–0.5)	0.776 (0.715–0.830)	89% (80.4–94.2)	72.5% (67.2–77.3)
HE4	54.8% (44.7–64.6)	90.1% (83.0–94.9)	5.5 (3.1–10.0)	0.5 (0.4–0.6)	0.724 (0.660–0.783)	83.8% (74.2–90.3)	68% (63.1–72.6)
CEA	55.8% (45.7–65.5)	83.8% (75.6–90.1)	3.4 (2.2–5.4)	0.5 (0.4–0.7)	0.698 (0.632–0.758)	76.3 % (67.1–83.6)	66.9% (61.6–71.8)
CYFRA21-1	48.1% (38.2–58.1)	67.6% (58.0–76.1)	1.5 (1.1–2.1)	0.8 (0.6–1.0)	0.578 (0.509–0.645)	58.1% (49.8–68.0)	58.1% (52.6–63.5)
NSE	32.7% (23.8–42.6)	77.5% (68.6–84.9)	1.5 (0.9–2.3)	0.9 (0.7–1.0)	0.551 (0.482–0.619)	57.6% (46.6–67.9)	55.1% (51.0–59.2)
*Biomarker combination*							
CEACAM6 + HE4	66.4% (56.4–75.3)	93.7% (87.4–97.4)	10.5 (5.1–21.8)	0.4 (0.3–0.5)	0.800 (0.740–0.851)	90.8% (82.6–95.3)	74.8% (69.3–79.6)
CEACAM6 + CEA	55.9% (43.8–63.7)	91.9% (85.2–96.2)	6.6 (3.5–12.7)	0.5 (0.4–0.6)	0.729 (0.664–0.787)	86.2% (76.4–92.3)	68.0% (63.2–72.5)
HE4 + CEA	49.1% (39.1–50.0)	95.5% (89.8–98.5)	10.9 (4.5–26.2)	0.5 (0.4–0.6)	0.723 (0.658–0.781)	91.1% (80.9–96.1)	66.7% (62.3–70.8)
CEACAM6 + HE4 + CEA	69.2% (59.4–77.9)	94.5% (88.6–98.0)	12.8 (5.8–28.2)	0.3 (0.2–0.4)	0.819 (0.761–0.868)	92.3% (94.5–96.4)	76.6% (71.0–81.5)
CEA + CYFRA21-1 + NSE	33.7% (24.7–43.6)	98.2% (93.6–99.8)	18.7 (4.6–75.7)	0.7 (0.6–0.8)	0.659 (0.592–0.601)	94.6% (81.2–98.6)	61.2% (57.9–64.5)

## 4. Discussion

The gold standard for the diagnosis of MPE is pathological cytology [25]. However, the method has a low sensitivity. Therefore, researchers have focused on the development of MPE biomarkers and the establishment of corresponding diagnostic models.

A previous study identified CD66c as a potential tumour marker for differentiating MPE associated with LUAD from benign effusions [15]. In our study, we focused on evaluating the diagnostic efficacy of CEACAM6 for both MPE and LC-MPE. We found an AUC value of 0.862 (0.804–0.909) for MPE and 0.922 (0.869–0.958) for LC-MPE in the test cohort, and 0.811 (0.722–0.899) for MPE and 0.866 (0.778–0.929) for LC-MPE in the validation cohort.

Although some studies have reported that HE4 has diagnostic value for MPE [20] and LC-MPE [21], our study is the first to investigate the combination of CEACAM6 and HE4 for diagnosing MPE and LC-MPE, and it demonstrated strong diagnostic efficacy. In the test cohort, the combination of CEACAM6 and HE4 significantly increased the AUC value to 0.938 for MPE and 0.967 for LC-MPE, outperforming any single biomarker. CEA is the most widely used biomarker for MPE in clinical practice [26]. Previous research has shown that CEA has a higher sensitivity than CA19-9, and CYFRA21-1 [15], which was also discovered in our study. Although the sensitivity of either CEACAM6 (73.2%) or HE4 (61.8%) was lower than that of CEA (75.9%), the specificity of CEA (88.1%) was much lower than that of CEACAM6 (93.2%) and HE4 (96.6%). Consequently, we explored the combined use of CEACAM6, HE4 and CEA. Adding CEA to the combination of CEACAM6 and HE4 slightly improved the AUC value to 0.941 for MPE and 0.970 for LC-MPE, surpassing the diagnostic performance of any single biomarker or any other two-biomarker combination in this study. We further validated our findings through an independent validation cohort. As expected, CEACAM6 and HE4 continued to show favourable diagnostic efficacy for MPE and LC-MPE. The combination of CEACAM6 and HE4 yielded AUC values of 0.834 for MPE and 0.876 for LC-MPE, while the addition of CEA improved AUC values to 0.852 for MPE and 0.925 for LC-MPE. Taken together, the combination of CEACAM6, HE4 and CEA was an important and valuable biomarker panel for MPE and LC-MPE diagnosis.

Considering the limited sensitivity of PE cytology, which leads to numerous false negatives in clinical practice, we excluded cytology positive cases and, for the first time, used ROC curve analysis to assess the diagnostic value of CEACAM6 and HE4 in cytology negative cases. As anticipated, our results demonstrated that the combination of CEACAM6 and HE4 provided satisfactory diagnostic performance for MPE. The combination achieved an AUC of 0.800 (0.740–0.851) for diagnosing MPE, and the addition of CEA further increased the AUC to 0.819 (0.761–0.868) (*p* = .04). Collectively, the combination of CEACAM6, HE4 and CEA proved to be a crucial biomarker panel for diagnosing MPE in cytology-negative cases.

Our previous study confirmed the diagnostic value of HE4 and CEACAM6 in CSF for LC-LM patients [22,23]. The sensitivity, specificity and AUC values for differentiating LC-LM from non-LM or normal controls were significantly higher than those for differentiating LC-MPE and BPE. CEACAM6 and HE4 in CSF are primarily derived from tumour cells. However, CEACAM6 is also exprexssed on neutrophils [26], which are frequently present in PE and may affect CEACAM6 levels, potentially reducing diagnostic accuracy. In addition, the composition of PE is more complex than that of CSF, with various factors influencing the levels of CEACAM6 and HE4. A comparative analysis of CEACAM6 and HE4 levels in CSF versus paired PE would be of interest and warrants further investigation.

Nevertheless, our study has several limitations. First, all the data analysed in this study came from a single centre with a relatively small sample size, which may limit the generalizability of our findings. Second, we did not monitor the dynamic changes in CEACAM6 and HE4 levels in PE during treatment, nor did we assess their correlation with therapeutic outcomes. Additionally, we used ELISA technique to detect CEACAM6, which may have lower sensitivity and specificity compared to chemiluminescence assays.

## 5. Conclusions

In conclusion, pleural CEACAM6 and HE4 are promising biomarkers for MPE. Their combination further enhances diagnostic accuracy, offering a valuable tool for differentiating MPE from BPE, especially in challenging cases with cytology-negative PE. Measuring pleural CEACAM6 and HE4 may facilitate detection of MPE, potentially improving patient prognosis.

## Supplementary Material

supplementary material.docx

Figure S1.tif

Figure S2.tif

## Data Availability

The data that support the findings of this study are available on request from the corresponding author. The data are not publicly available due to privacy or ethical restrictions.
