# Supplementary material for: Diagnostic value of CEACAM6 and HE4 in pleural fluid for malignant pleural effusion
Source: Ann Med. 2025 Apr 15;57(1):2489748. doi: 10.1080/07853890.2025.2489748 (PMC12001857; doi:10.1080/07853890.2025.2489748)
Supplement: supplementary material.docx [file IANN_A_2489748_SM7268.docx]

Supplementary Material

Fig. S1


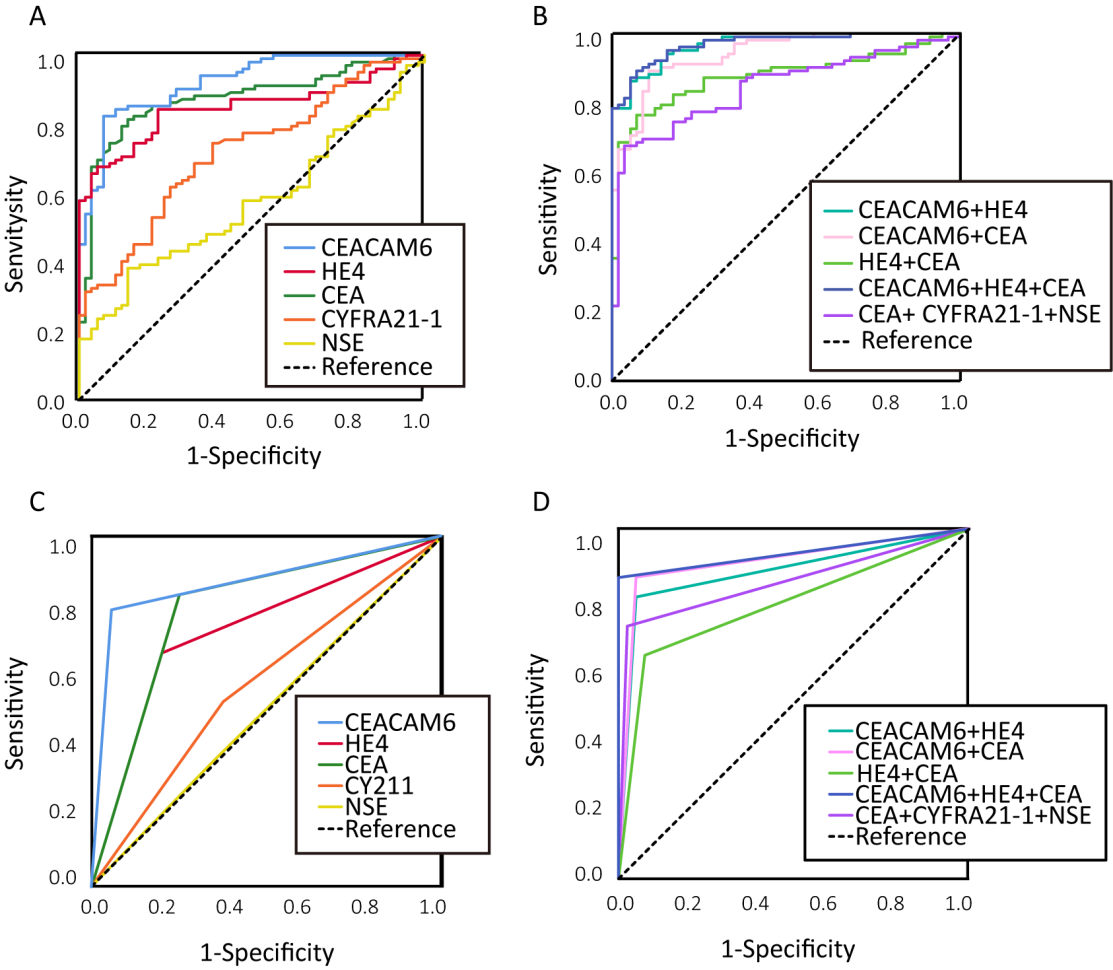


**Figure S1**. The ROC curve for differentiating LC-MPE from BPE in both test cohort and validation cohort. (A) The ROC curve of a single biomarker in test cohort. (B) The ROC curve of the combination of the biomarkers in test cohort. (C) The ROC curve of a single biomarker in validation cohort. (D) The ROC curve of the combination of the biomarkers in validation cohort.

Fig.S2


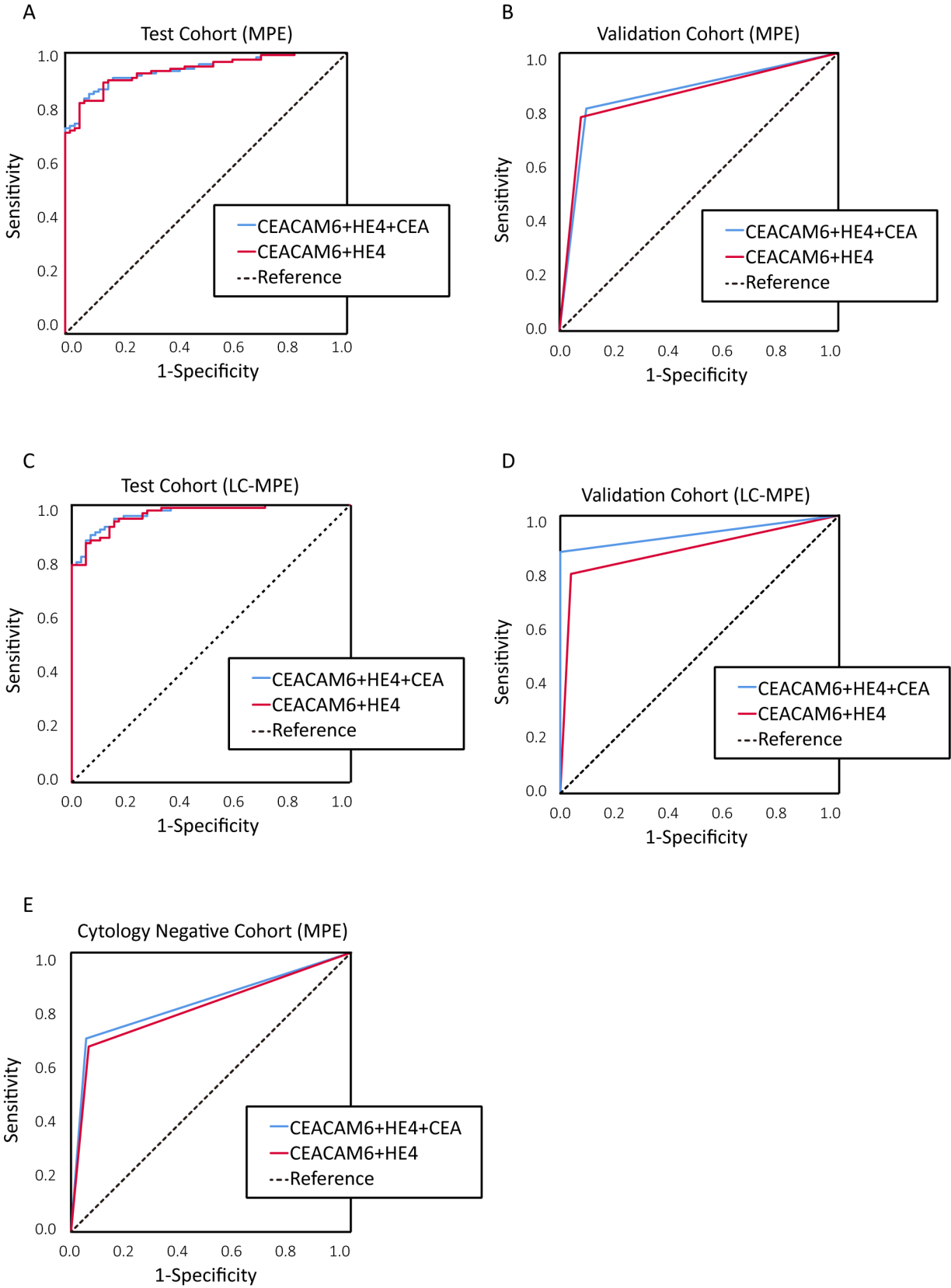


**Figure S2.** Comparison of ROC curves between the combined use of CEACAM6 and HE4 and the combination of CEACAM6, HE4 and CEA. (A-B) The ROC curve of test and validation cohort in diagnosing MPE. (C-D) The ROC curve of test and validation cohort in diagnosing LC-MPE. (E) The ROC curve of cytology negative cohort in diagnosing MPE.

**Table S1.** Comparison of ROC curves between the combined use of CEACAM6 and HE4 and the combination of CEACAM6, HE4 and CEA in different cohort

| **CEACAM6+HE4** vs **CEACAM6+HE4+CEA** | **Difference between areas** | **SE** | **95% CI** | **z value** | p**-value** |
| --- | --- | --- | --- | --- | --- |
| **Test Cohort (MPE)** | 0.0024 | 0.0036 | -0.0046 to 0.0094 | 0.678 | 0.50 |
| **Validation Cohort (MPE)** | 0.0058 | 0.0181 | -0.0298 to 0.0413 | 0.318 | 0.75 |
| **Test Cohort (LC-MPE)** | 0.0032 | 0.0037 | -0.0040 to 0.0103 | 0.861 | 0.39 |
| **Validation Cohort (LC-MPE)** | 0.0491 | 0.0278 | -0.0054 to 0.1040 | 1.766 | 0.08 |
| **Cytology** **Negative Cohort (MPE)** | 0.0189 | 0.0094 | 0.0005 to 0.0373 | 2.014 | 0.04 |

**Table S2**. Diagnostic efficacy of individual pleural biomarkers and their combinations for identifying LC-MPE in test cohort

| **Variables** | **cut-off value** | **Sensitivity (95%CI)** | **Specificity (95%CI)** | **+LR (95%CI)** | **-LR (95%CI)** | **AUC (95%CI)** | **PPV (95%CI)** | **NPV (95%CI)** |
| --- | --- | --- | --- | --- | --- | --- | --- | --- |
| **Single biomarker** | |  |  |  |  |  |  |  |
| CEACAM6 | 17.65 (ng/mL) | 82.7% (73.6-89.2) | 93.2% (83.5-98.1) | 12.2 (4.7-31.4) | 0.2 (0.1-0.3) | 0.922 (0.869-0.958) | 95.5% (91.0-97.8) | 75.3% (66.7-82.3) |
| HE4 | 780.10 (pmol/L) | 67.7% (57.7-76.6) | 94.9% (85.9-98.9) | 13.3 (4.4-40.4) | 0.3 (0.3-0.5) | 0.847 (0.782-0.899) | 95.8% (90.8-98.2) | 63.0% (56.1-69.3) |
| CEA | 2.74 (ng/mL) | 81.4% (72.4-88.4) | 86.4% (75.0-94.0) | 6.0 (3.1-11.5) | 0.2 (0.1-0.3) | 0.877 (0.817-0.924) | 91.2% (86.3-94.5) | 72.9% (64.0-80.2) |
| CYFRA21-1 | 23.58 (ng/mL) | 68.6% (58.7-77.5) | 67.8% (54.4-79.4) | 2.1 (1.4-3.2) | 0.5 (0.3-0.6) | 0.722 (0.646-0.790) | 78.7% (73.0-83.4) | 55.6% (47.7-63.2) |
| NSE | 15.1 (ng/mL) | 38.4% (28.8-48.4) | 86.0% (74.2-93.7) | 2.7 (1.4-5.4) | 0.7 (0.6-0.9) | 0.571 (0.497-0.650) | 82.5% (73.3-89.0) | 44.6% (10.4-48.9) |
| **Biomarker combination** | |  |  |  |  |  |  |  |
| CEACAM6+HE4 | - | 86.3% (78.0-92.3) | 94.9% (85.9-98.9) | 17.0 (5.6-51.2) | 0.1 (0.09-0.2) | 0.967 (0.926-0.989) | 96.7% (92.7-98.6) | 80.0% (71.1-86.7) |
| CEACAM6+CEA | - | 89.2% (81.5-94.5) | 89.8% (79.2-96.2) | 8.8 (4.1-18.8) | 0.1 (0.07-0.2) | 0.942 (0.894-0.973) | 93.8% (89.5-96.4) | 82.8% (79.3-89.4) |
| HE4+CEA | - | 76.5% (67.0-84.3) | 93.2% (83.5-98.1) | 11.3 (4.4-29.2) | 0.3 (0.2-0.4) | 0.876 (0.815-0.923) | 95.1% (90.4-97.6) | 69.6% (61.7-76.6) |
| CEACAM6+HE4+CEA | - | 89.2% (81.5-94.5) | 93.2% (83.5-98.1) | 13.2 (5.1-34.0) | 0.1 (0.07-0.2) | 0.970 (0.930-0.990) | 95.8% (91.7-97.9) | 83.3% (74.1-89.8) |
| CEA+CYFRA21-1+NSE | - | 67.7% (57.7-76.6) | 96.5% (87.9-99.6) | 19.3 (4.9-75.7) | 0.35 (0.3-0.4) | 0.845 (0.779-0.897) | 97.1% (92.3-98.9) | 63.3% (56.5-69.6) |

**Table S3**. Diagnostic efficacy of individual pleural biomarkers and their combinations for identifying LC-MPE in validation cohort

| **Variables** | **Sensitivity (95%CI)** | **Specificity (95%CI)** | **+LR (95%CI)** | **-LR (95%CI)** | **AUC (95%CI)** | **PPV (95%CI)** | **NPV (95%CI))** |
| --- | --- | --- | --- | --- | --- | --- | --- |
| **Single biomarker** |  |  |  |  |  |  |  |
| CEACAM6 | 79.0% (62.7-90.4) | 94.2% (84.1-98.8) | 13.7 (4.5-41.6) | 0.2 (0.1-0.4) | 0.866 (0.778-0.929) | 90.9% (76.7-96.8) | 86.0% (76.7-91.9) |
| HE4 | 68.4% (51.3-82.5) | 82.7% (69.7-91.8) | 4.0 (2.4-7.4) | 0.4 (0.2-0.6) | 0.756 (0.654-0.840) | 74.3% (60.6-84.5) | 78.2% (68.8-85.3) |
| CEA | 84.2% (68.7-94.0) | 69.2% (54.9-81.3) | 2.7 (1.8-4.2) | 0.2 (0.1-0.5) | 0.767 (0.666-0.850) | 66.7% (56.5-75.5) | 85.7% (73.8-92.7) |
| CYFRA21-1 | 52.8% (35.5-69.6) | 62.8% (46.7-77.0) | 1.4 (0.9-2.3) | 0.8 (0.5-1.1) | 0.578 (0.461-0.688) | 54.3% (42.0-66.1) | 61.4% (51.2-70.6) |
| NSE | 38.8% (22.5-55.2) | 64.4% (48.8-78.1) | 1.1 (0.6-1.9) | 1.0 (0.7-1.3) | 0.511 (0.398-0.623) | 46.7% (33.1-60.8) | 55.8% (47.5-63.7) |
| **Biomarker combination** |  |  |  |  |  |  |  |
| CEACAM6+HE4 | 79.0% (62.7-90.4) | 96.2% (86.8-99.5) | 20.5 (5.2-80.7) | 0.2 (0.1-0.4) | 0.876 (0.789-0.936) | 93.8% (79.2-98.3) | 86.2% (77.1-92.1) |
| CEACAM6+CEA | 86.8% (71.9-95.6) | 92.3% (81.5-97.9) | 11.3 (4.4-29.2) | 0.1 (0.06-0.3) | 0.896 (0.813-0.950) | 89.2% (76.1-95.5) | 90.6% (80.9-95.6) |
| HE4+CEA | 65.8% (48.6-80.4) | 92.3% (81.5-97.9) | 8.6 (3.2-22.5) | 0.4 (0.2-0.6) | 0.790 (0.692-0.869) | 86.2% (70.3-94.3) | 78.7% (70.2-85.2) |
| CEACAM6+HE4+CEA | 86.8% (71.9-95.6) | 98.1% (89.7-100) | 45.2 (82.5-99.6) | 0.1 (0.06-0.3) | 0.925 (0.849-0.970) | 97.1% (82.5-99.6) | 91.1% (81.8-95.9) |
| CEA+CYFRA21-1+NSE | 72.2% (54.8-85.8) | 97.5% (86.8-99.9) | 28.9 (4.1-202.2) | 0.3 (0.2-0.5) | 0.849 (0.748-0.921) | 96.3% (78.8-99.5) | 79.6% (69.7-86.9) |

**Table S4.** Composition of the cytology negative cohort

|  | **MPE** | **BPE** |
| --- | --- | --- |
| **Test Cohort** | 60 | 59 |
| **Validation Cohort** | 40 | 52 |
