# Supplementary figures and images for: Diagnostic value of CEACAM6 and HE4 in pleural fluid for malignant pleural effusion
Source: Ann Med. 2025 Apr 15;57(1):2489748. doi: 10.1080/07853890.2025.2489748 (PMC12001857; doi:10.1080/07853890.2025.2489748)

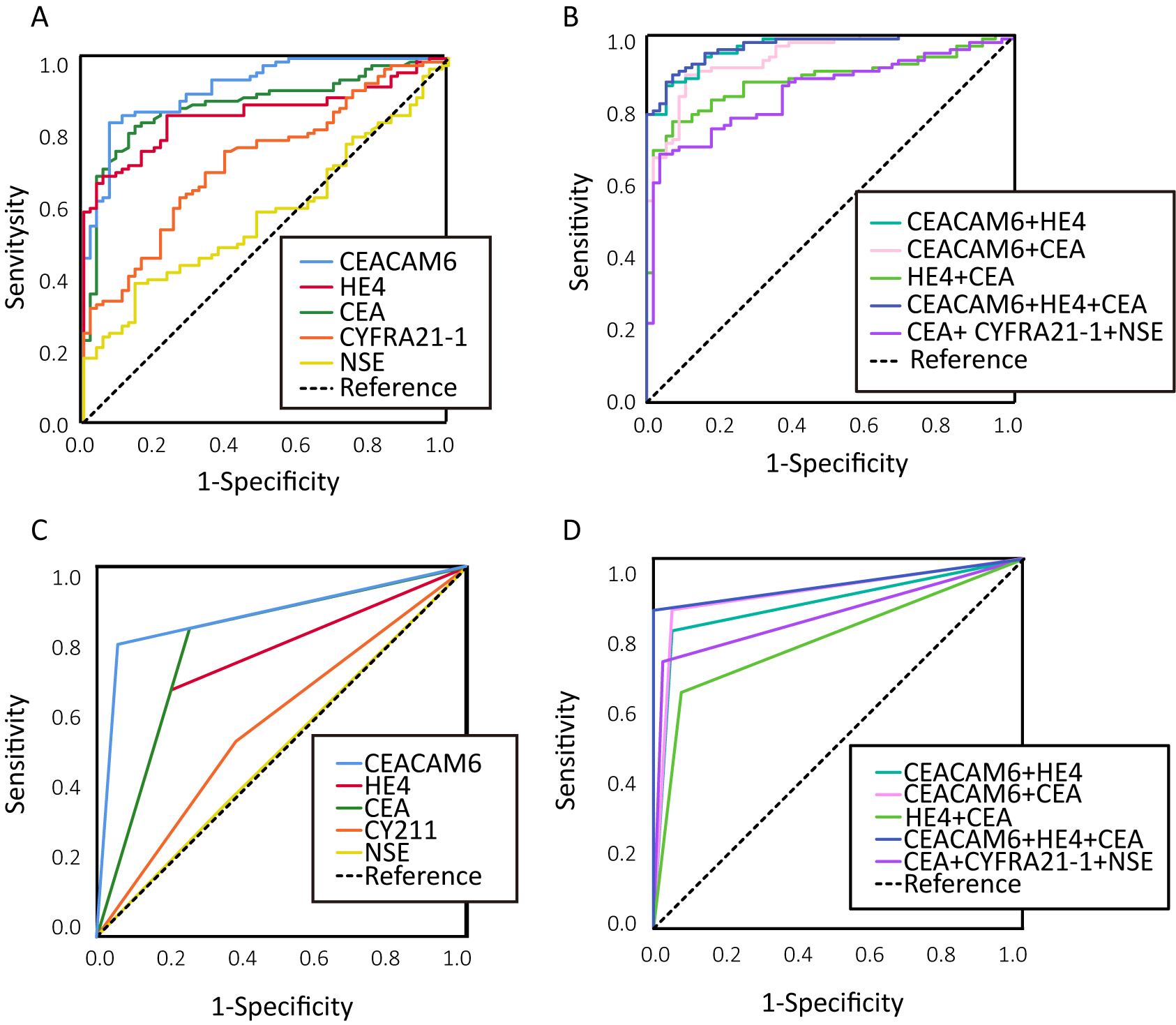

Supplement: Figure S1.tif [file IANN_A_2489748_SM7267.tif]

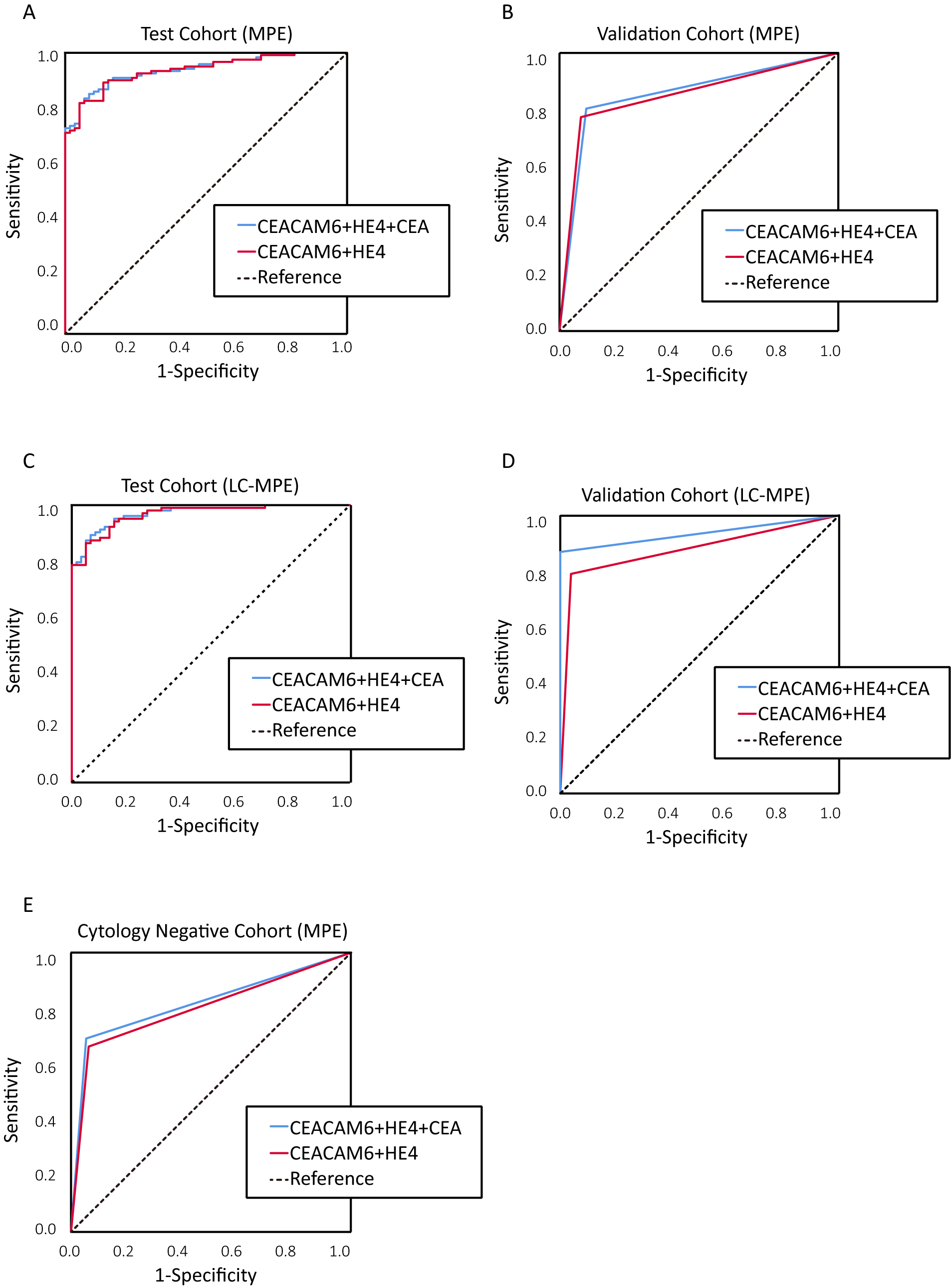

Supplement: Figure S2.tif [file IANN_A_2489748_SM7266.tif]
